# Worldwide productivity and research trend of publications concerning electroactive materials and spinal cord injury: A bibliometric study

**DOI:** 10.3389/fbioe.2023.1094059

**Published:** 2023-02-27

**Authors:** Sirui Liu, Lin Song, Weishu Dai, Mengdie Liu, Huijing Zhang, Xueyan Zhang, Hongyu Li, Xiu Liu, Yan Lv, Ying Hu

**Affiliations:** ^1^ Beijing Institute of Dental Research, Beijing Stomatological Hospital, Capital Medical University, Beijing, China; ^2^ Department of Periodontology, Beijing Stomatological Hospital, Capital Medical University, Beijing, China; ^3^ The Third Medical Center of PLA General Hospital, Beijing, China; ^4^ The First Medical Center of PLA General Hospital, Beijing, China

**Keywords:** bibliometrix, Citespace, electroactive materials, spinal cord injury, biomaterials

## Abstract

**Purpose:** We investigated the current state and trends in the area during the previous 10 years using bibliometric approaches to evaluate the global scientific output of research on electroactive materials and spinal cord injury.

**Methods:** Studies on spinal cord injury in electroactive materials that were published between 2012 and 2022 were located using the Web of science (WOS) datebase. The software programs bibliometrix R-package and CiteSpace were used to do quantitative analyses of annual publications, nation, author, institution, journal source, co-cited references, and keywords. The studies were categorized by the research’s main points using a qualitative analysis, and publications having more than 10 citations each year.

**Results:** In the final analysis, 1,330 relevant papers or reviews were included. There is an increased tendency in both the average annual citation rate and the number of publications in the discipline. The United States and the University of Toronto are the countries and institutions that have contributed the most to this discipline, respectively. The majority of authors are from the China and United States. Zhang Y is the author with the most published articles and holds the top position in the cited author h-index species. The journal with the highest number of published articles is “Disability and rehabilitation”; the journal is divided into four main areas including physics, materials, chemistry, molecular, and biology. The keyword analysis revealed a shift in research hotspots from schwann cell, fracture, and urinary disorders to carbon-based materials, functional recovery, and surgery. Analysis of qualitative data revealed that the role and mechanism of injectable conductive hydrogels in spinal cord healing after damage is a hot topic of current study, with the mechanism primarily focusing on the inhibition of oxidative stress (Nrf2) and apoptosis (Casepase 3).

**Conclusion:** Our bibliometric analysis indicates that research on electroactive materials for spinal cord injury remains an active field of study. Moreover, contemporary research is concentrated on carbon-based materials, functional rehabilitation, and surgery.

## 1 Introduction

The number of spinal cord injuries caused by trauma and other causes surpasses 18,000 annually in United States (Liu N. K. et al., 2021; Ameer and Tessler, 2022). About 45% of these patients have spinal motor neuron lesions, whereas 95% of the patients with lumbar or sacral injuries have spinal cord damage (Liu N. K. et al., 2021). Spinal cord injuries are classified as primary or secondary (Yuan et al., 2021). Primary spinal cord injury resulting in axonal disruption, neural tissue destruction and neuronal death, with partial functional repair primarily through surgical decompression and spinal fixation (Antar et al., 2018; Huang et al., 2022). Secondary injuries are long-term progressive injuries that occur after the primary injury resulting in lesion spread and stage destruction, including neuronal/axonal loss, formation of a neuroregenerative inhibitory microenvironment and glial scarring (Doherty et al., 2002; Wang et al., 2022). However, the death of motor neurons, interneurons, and glial cells at the site of the lesion owing to subsequent damage predisposes to a decrease in muscle tissue mass and fiber cross-sectional fiber diameter, resulting in a loss of function (Liu X. et al., 2021; Fouad et al., 2021). The loss of function after spinal cord injury is mainly due to the limited ability of damaged neurons in the central nervous system (CNS) to axonize and re-establish functional connections (Zheng and Tuszynski, 2023). The only medications now available for clinical therapy involve large dosages of methylprednisolone (MP); nevertheless, systemic administration of high doses of MP can result in extremely severe adverse effects (Karabey-Akyurek et al., 2017). Therefore, a medication delivery channel must have delayed drug release and excellent biocompatibility.

Electroactive biomaterials can convey electrical impulses directly *via* regulated electrical potential, with enhancing biocompatibility and mitigating or reducing the side effect of drug (Tandon et al., 2018; Li et al., 2023). This family consists mostly of conductive polymers (CPs), piezoelectric materials, and nanomaterials based on carbon. CPs offer straightforward production methods and advantageous electrical and machinery characteristics comparable to metals and semiconductors (Ning et al., 2018). Among them, hydrogels are widely used for SCI repair due to their hydrophilicity and biocompatibility, such as drug delivery, lubrication, conductive coating of nerve electrode, etc., (Wang et al., 2022). Piezoelectric materials may generate an electrical charge in reaction to mechanical deformation and undergo mechanical deformation in response to an applied electric field; simultaneously, piezoelectric effects can modify cellular function and regulate the growth and remodeling of bone tissue and axon guidance (Tandon et al., 2018; Dai et al., 2022; Xiong et al., 2022). In particular, polylactic acid (PLLA) has good piezoelectric properties and the ability to carry conductive polymers that mimic the extracellular matrix, maintain cell stemness and promote axon guidance (Dai et al., 2022). Carbon-based nanomaterials with superior biocompatibility and mechanical characteristics can influence cell behavior to achieve gradual drug release while altering cell proliferation, differentiation, and possible antibacterial action against Gram-negative and Gram-positive bacteria strains (Dong et al., 2017; More et al., 2017). Graphene is a monolayer of SP2 in a two-dimensional arrangement of hybridized carbon atoms (2D) honeycomb lattice. Graphene-based materials (GBM) play a neuroprotective role by modulating endogenous inflammatory responses to counteract cell death and limit the spread of damage, while GBM can act as a scaffold to build effective bridging networks and promote the survival and differentiation of neural stem cells (NSCs) for neuroregeneration (Girao et al., 2022). Therefore, these materials may be employed as scaffolds to promote cell growth and tissue regeneration, as well as scaffolds and drug carriers to repair (restore) spinal cord injuries, garnering the interest of medical researchers throughout the world. Using bibliometrics, we conducted a quantitative and qualitative examination of the current condition of the subject in order to highlight previous and present research hotspots from several viewpoints.

In bibliometrics, quantitative and qualitative analysis is applied to journals and publications, as well as their citation counts over time and distribution patterns within a particular topic, field, institution, and nation (Nielsen et al., 2017; Boudry et al., 2018). Bibliometrics can assist in identifying research topics, planning research paths, and anticipating research trends (Fu et al., 2013). In bibliometrics, co-citation and co-linearity are used to identify the research base and research hotspots, respectively. In this study, we use bibliometrics to detect worldwide research trends, evaluate successes in the fields of electroactive materials and spinal cord injury restoration, and foresee potential future hotspots.

## 2 Materials and methods

### 2.1 Search strategy

**FIGURE 1:**
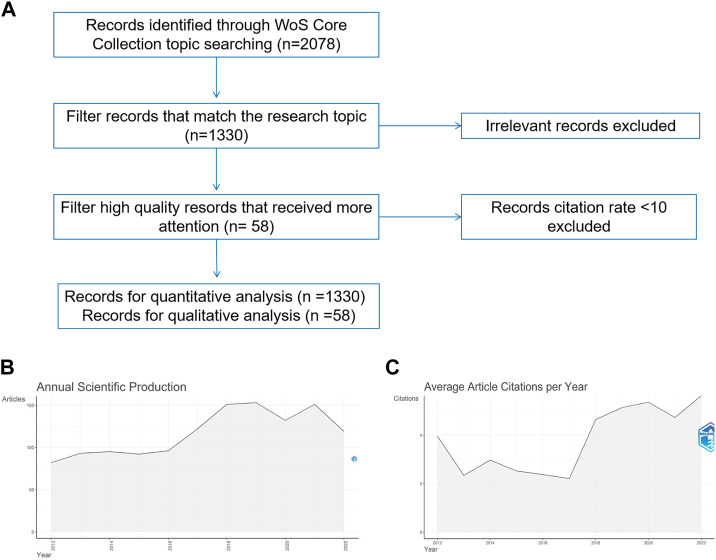
Overall situation **(A)** Literature screening graph **(B)** Year-by-year publication growth graph **(C)** Year-by-year citation growth graph.

The Web of Science (WOS) core database was searched on 16 October 2022, and all data were acquired. According to the procedure for conducting research, the search formula was TS = (“spinal cord injury” or “spinal cord injuries”), TS = (electroactive, conductive, piezoelectric, or carbon-based) or (material or biomaterial, hydrogel, scaffolds, polymers, or ceramics) or polypyrrole or PPy, polyaniline, PANi, aniline oligomer, polyvinylidene fluoride, PVDF, Kynoar,l-polylactic acid, polylactic-L acid, PLLA. For the period from 2012 to 2022. The final selection includes 1,330 published works on the subject of electroactive materials and spinal cord damage. The extracted data includes reference type, journal title, publication date, author name and affiliation, and abstract. Only original articles and reviews were included in our analysis; all other types of papers were eliminated due to stringent screening. Two writers (LSR and LY) completed the study selection procedure; if a dispute developed during the selection process, the experienced corresponding author made the choice to include the publication in our study. Figure 1A depicts information on the selection in great detail. In addition, qualitative analysis included bibliometric screening of articles with an average yearly citation count greater than ten.

### 2.2 Bibliometric analysis and visualization

Bibliometrix is a scientific bibliometric software developed by Federico II University of Naples, Italy, using the R programming language. Consistent with past research, the R 4.0.3 bibliometrix program is used to automatically transform and evaluate publication data (Huang et al., 2022; Xiong et al., 2022). The research contains the number of publications each year, the number of publications by nation, an analysis of authors and institutions, an analysis of journals, co-cited references, and important terms. Indicators used to evaluate the quality of authors’ publications include the number of publications, citations in the field of research, and the H-index of author citations (Xiong et al., 2022). The Publication H Index is utilized to assess a scientist’s research output and evaluate his field effect (Brahler and Decker, 2005).

Citespace is a scientometric research tool created by the School of Computing and Intelligence at Drexel University in the United States (Xiong et al., 2022). Citespace is utilized for co-authorship analysis of countries/authors/institutions, co-citation analysis of journals/references, contribution analysis of keywords, and visualization employing grids, overlays, etc.

## 3 Results

### 3.1 General description

On 16 October 2022, the WOS core repository had 2078 articles pertaining to electroactive materials and spinal cord damage. In order to investigate the most significant tendencies that have emerged during the past 10 years, we focused our research on the years 2012–2022.1,330 publications are released between 2012 and 2022, with 1,143 papers (85.93%) and 187 reviews (14.06%) respectively (Figure 1A). Despite a modest decline in the number of publications in 2020, there has been an overall rising tendency in the number of yearly publications in the subject since the turn of the 20th century (Figure 1B). The total number of citations for these 1,330 papers was 1,286, as seen in Figure 1C, which depicts average yearly citation rates. The average number of citations per year is calculated by dividing the total number of citations by the number of years after publication (Huang et al., 2022). The greater the average number of citations each year, the more the article serves as a foundation for study in its subject or as a research hotspot during the year (Xiong et al., 2022). The average article citation rate exhibited a little downward trend from 2012 to 2014, a modest increase in the average annual citation rate after 2017, and a quick upward fluctuation after 2018, reaching a high average annual citation rate of 4.72 in 2022. According to the findings, the application of electroactive materials in the treatment of spinal cord injuries is gradually becoming a new field of study focus.

### 3.2 Country and regional analysis

**FIGURE 2:**
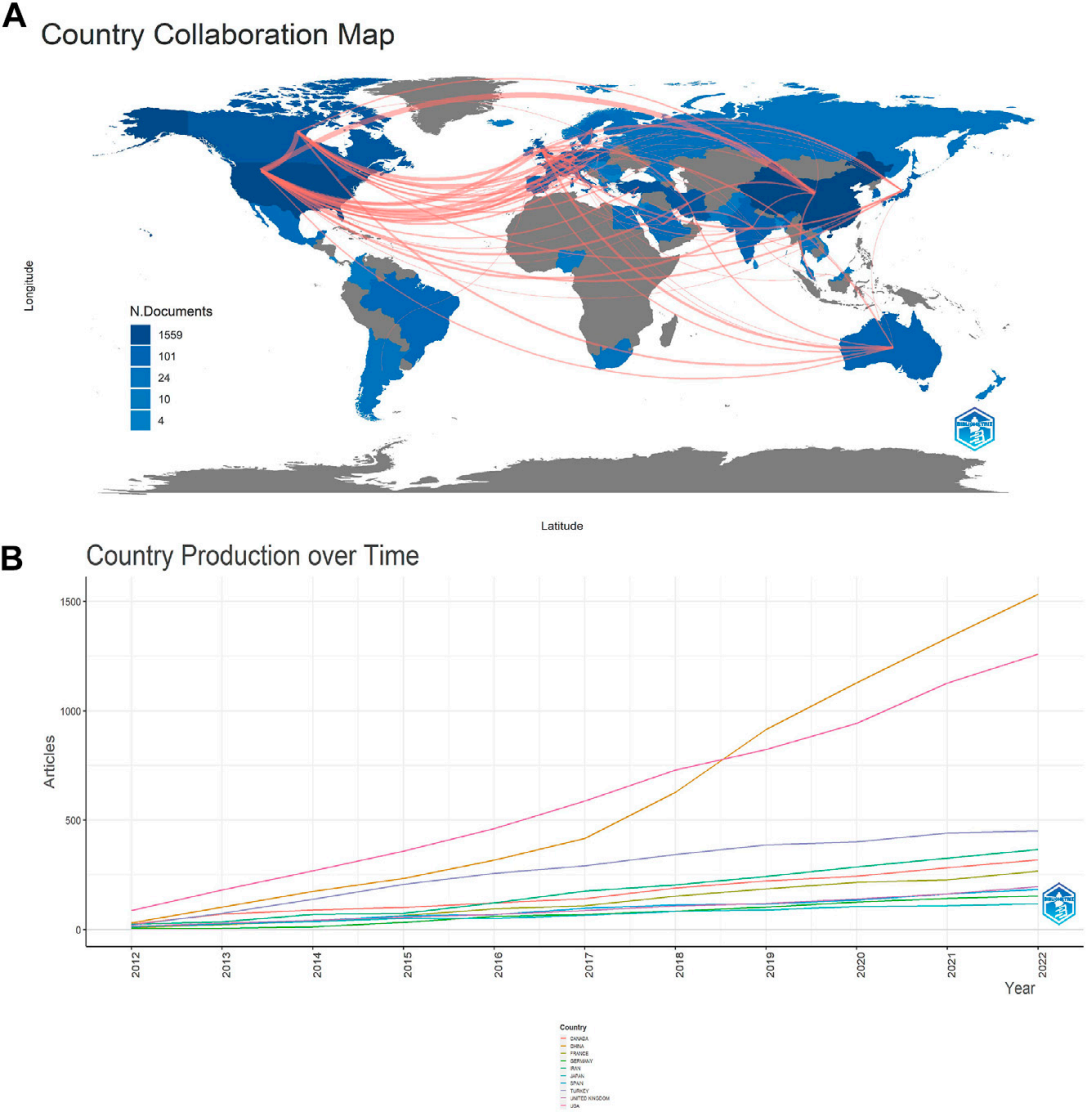
Analysis of the distribution of countries and publications. **(A)** Map of cooperation between SCI and electroactive materials countries (gray, no relevant publications in the country; blue, the country has relevant publications; blue depth, number of publications; red line, cooperation between connected countries, line thickness, frequency of cooperation); **(B)** Change in the number of publications in the 10 countries with the most publications from 2012 to 2022.

The corresponding authors of the 1,330 articles were divided throughout 67 nations or regions (Figure 2A). Average article citations for major participating countries and top five affiliations are listed in Tables 1, 2. Figure 2B depicts the ten nations with the largest number of publications, where the number of articles connected to the United States climbed consistently over time, and the number of articles related to China has progressively overtaken that of the United States since 2018. In addition, publications from the United States (6,241) and China (4,556) were mentioned far more frequently than those from other nations, demonstrating that research in these nations is at the forefront relative to other nations. The top ten nations with high average article citation rates are listed in Table 1, along with their respective citation rates worldwide. On the other hand, the average citation rate of publications published in the United States of America is much greater than that of articles published in China, demonstrating the superior quality of articles published in the United States of America in this field.

**TABLE 1:** Average article citations for major participating countries.

Country	TC	Average article citations (%)
UNITED STATES	6,241	24.67
CHINA	4,556	12.66
CANADA	1,258	22.87
UNITED KINGDOM	840	18.67
IRAN	716	10.53
TURKEY	601	6.20
KOREA	475	19.79
FRANCE	467	9.53
GERMANY	415	12.97
ITALY	356	13.69

**TABLE 2:** Top five affiliation abbreviations and number of published articles.

Affiliation	Abbreviations	Articles
University of Toronto	UNIV TORONTO	86
Tehran University of Medical Sciences	UNIV TEHRAN MED SCI	63
Iran University of Medical Sciences	IRAN UNIV MED SCI	51
SunYat-sen University	SUN YAT SEN UNIV	50
Capital Medical University	CAPITAL MED UNIV	49

### 3.3 Authors and institutional analysis

**FIGURE 3:**
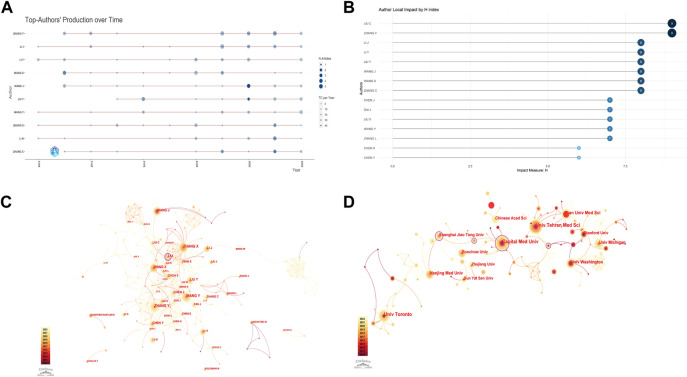
Visualization of active institutes and authors analysis. **(A)** Timeline distribution of top 10 most productive authors. Red line, temporal distribution of author-related publications; circle diameter, number of publications; light blue, total citations per year. **(B)** h-index of publications from different authors. **(C)** Cluster analysis of cooperation among authors. **(D)** Cluster analysis of cooperation institutes.

The top 10 authors with the highest total citations of papers published between 2012 and 2022 are shown in Figure 3A. Zhang Y is the author with the most publications with 24 articles (14.72% of all articles), followed by Li J (18, 11.04%); additionally, both authors have high total citations per year (Figure 3A light blue circles); the h-index of publications is highest for Liu C and Zhang Y had the highest index (9) followed by Li J (8), Li Y (8), Liu Y (8), Wang J (8), Wang X (8), Zhang C (8), and Liu (Figure 3B). The connecting lines between the authors are the co-authors who created the papers, and the findings indicate that WANG Y (Centrality = 0.15), ZHANG X (0.08), ZHANG Y (0.07), WANG J (0.07), and WANG X (0.07), are the five authors with the highest mediated centrality (0.06) (Figure 3C).

University of Toronto (28.76%) has the most publications among research institutes, followed by Tehran University of Medical Sciences (21.07%) and Iran University of Medical Sciences (17.06%) (Table 3). Capital Medical University (Centrality = 0.16), Shanghai Jiao Tong University (Centrality = 0.11), and Nanjing Medical Sciences (Centrality = 0.1) were the institutions having the closest collaboration with other universities, as determined by an enrichment study utilizing citespace (Figure 3D).

**TABLE 3:** Top 20 sources in this filed.

Sources	Articles
DISABILITY AND REHABILITATION	61
EUROPEAN REVIEW FOR MEDICAL AND PHARMACOLOGICAL SCIENCES	57
MEDICAL SCIENCE MONITOR	51
TURKISH NEUROSURGERY	42
JOURNAL OF UROLOGY	38
NEUROMODULATION	35
JOURNAL OF SURGICAL RESEARCH	22
ACTA BIOMATERIALIA	18
JOURNAL OF TISSUE VIABILITY	17
EUROPEAN SPINE JOURNAL	16
IRANIAN JOURNAL OF BASIC MEDICAL SCIENCES	16
NEUROUROLOGY AND URODYNAMICS	14
INTERNATIONAL JOURNAL OF NEUROSCIENCE	13
NEURAL REGENERATION RESEARCH	13
BIOMATERIALS	12
REGENERATIVE MEDICINE	12
TURKIYE FIZIKSEL TIP VE REHABILITASYON DERGISI-TURKISH JOURNAL OF PHYSICAL MEDICINE AND REHABILITATION	12
AMERICAN JOURNAL OF NEURORADIOLOGY	11
BJU INTERNATIONAL	11

### 3.4 Analysis of journals and related fields

According to Table 3, “DISABILITY AND REHABILITATION” has published 61 publications in this topic. With 57 articles, “EUROPEAN REVIEW FOR MEDICAL AND PHARMACOLOGICAL SCIENCES” is the second most published journal. However, biomaterials, radiography, and urology are also included. The majority of the articles focus on neurology and rehabilitation. It is crucial for researchers in the field of spinal cord injury repair to pick a target journal based on the research focus of these journals, which offers a sound theoretical foundation for the repair of spinal cord injuries with electroactive materials.

**FIGURE 4:**
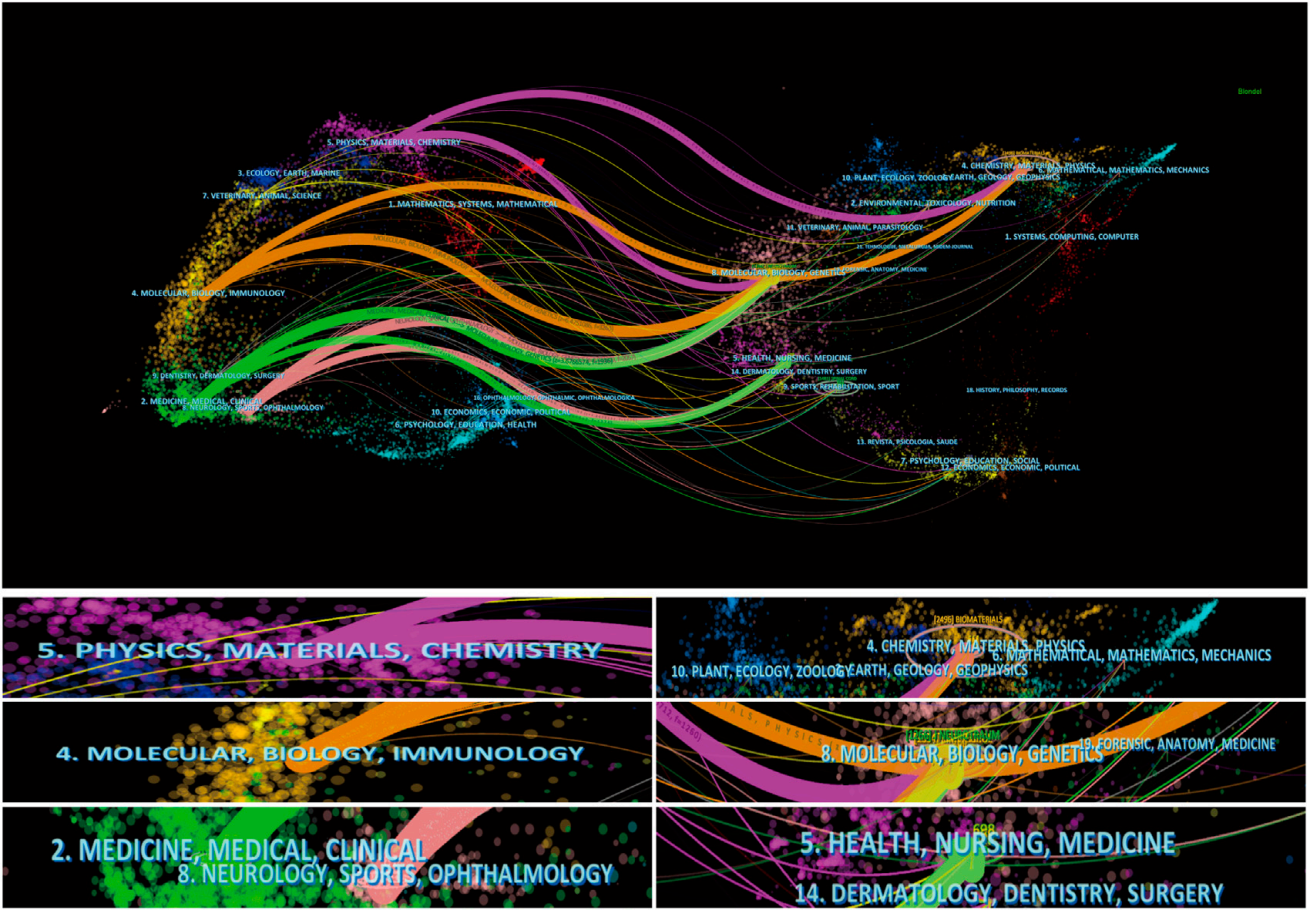
The dual-map overlay of articles citing (The left side were the right side were the cited journal, and the line path represents in the citation relationship).

The left side of Figure 4 illustrates the distribution of cited literature by journal, whereas the right side illustrates the location of cited literature by journal. Physic, materials, chemistry, and chemistry make up the four key categories of our study’s articles, as shown in Figure 4.

In addition, these publications’ references are mostly spread in the fields of chemistry, materials, physics, molecular biology, immunology, clinical medicine, neurology, sports medicine, and ophthalmology. We discovered that neurology, ophthalmology, physics, materials chemistry, molecular biology, and immunology are predominantly involved in the use of electroactive materials in spinal cord damage. The evolution of these disciplines is intrinsically linked to the evolution of the interaction between medical and scientific fields.

### 3.5 Total cited references

**FIGURE 5:**
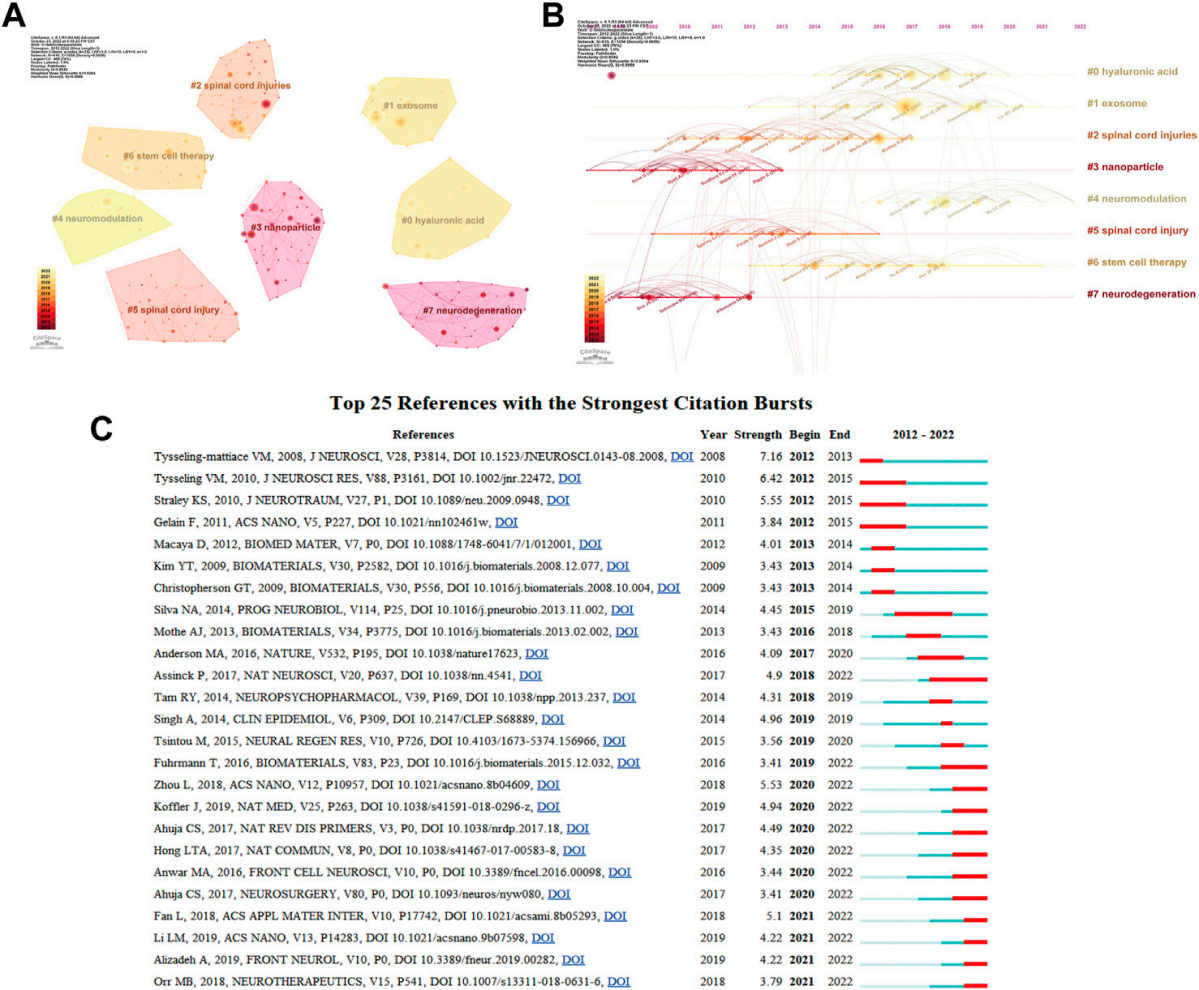
Visualization of co-cited reference analysis. **(A)** Cluster analysis of co-cited references; **(B)** Timeline distribution of the top 8 cluster; **(C)** Representative burst references among top 25 references with the strongest citation bursts.

Figure 5 depicts the most frequently mentioned works. Using citespace clustering analysis, it was shown that the topic phrases hyaluronic acid, exosome, spinal cord injuries, nanoparticle, neuromodulation, spinal cord injury, stem cell treatment, and neurodegeneration predominated in these publications (Figure 5A). Figure 5B depicts the development of this topic word throughout time. Figure 5C depicts the development of the referenced literature by displaying the 25 most-cited articles out of the total number of cited papers. The most often cited is the paper titled

Macrophage polarization: An opportunity for improved outcomes in biomaterials and regenerative medicine. The biggest number of citations reflects the significance of this literature in the subject of electroactive materials and spinal cord damage, which serves as the foundation for research in this field.

### 3.6 Keyword analysis

**FIGURE 6:**
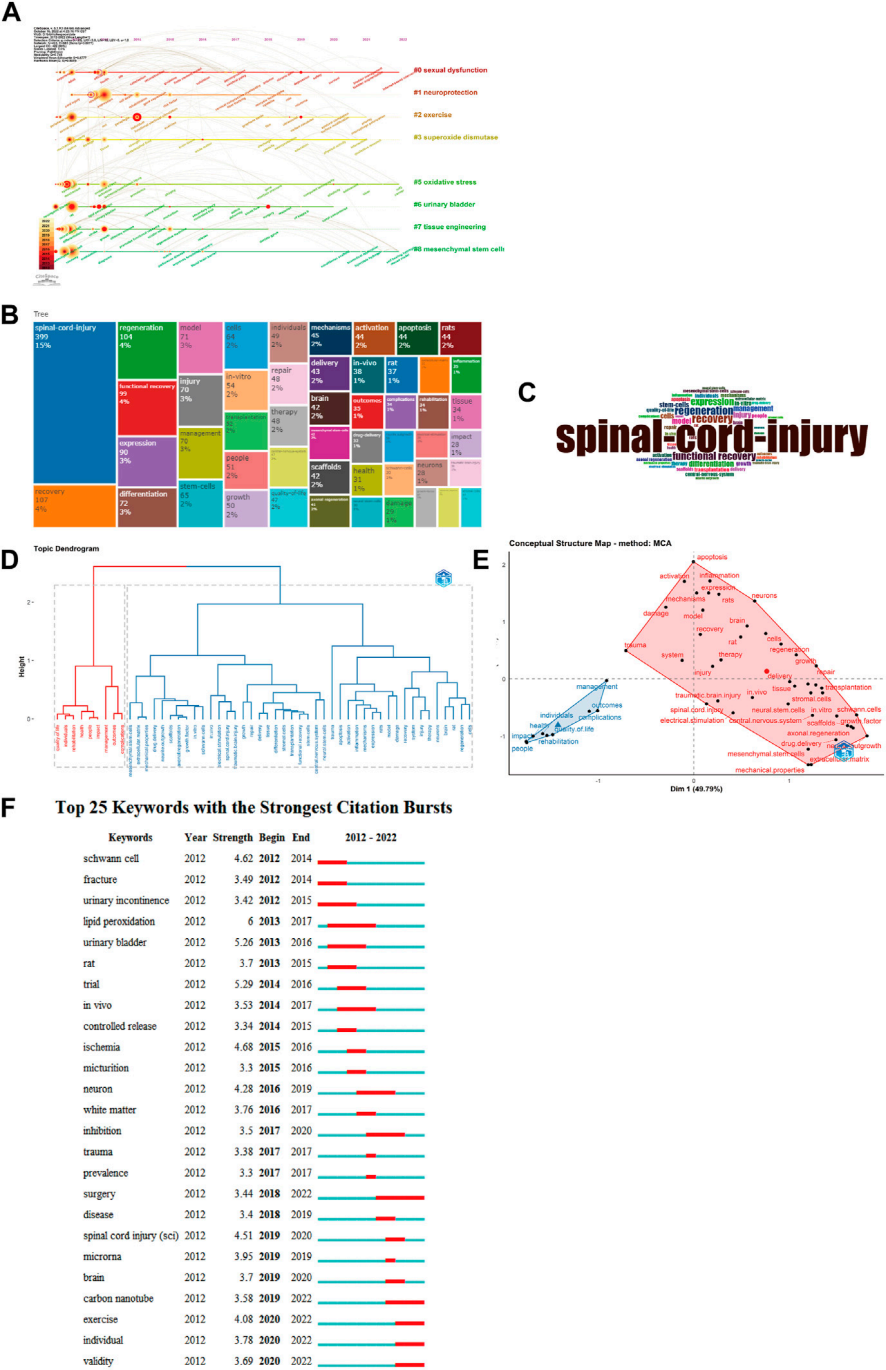
Visualization of keyword analysis. **(A)** Timeline distribution of cluster analysis of keyword; **(B)** World TreeMap; **(C)** World cloud; **(D)** Trend topics; **(E)** Conceptual structure map of common author keywords (Multilateral graphics: Topic clusters, keywords in the same cluster have similarity); **(F)**Representative burst keywords among top 25 keywords with the strongest citation bursts.

We obtained 10 grouped subject phrases using citespace clustering analysis, including sexual dysfunction, neuroprotection, etc (Figure 6A). The focus of studies on sexual dysfunction has shifted from “experienced adult” to “online intervention.” The Neuroprotection study eventually shifted its emphasis from “apoptosis” and “expression” to “microrna.” Figure 6B displays the frequency of keyword occurrence, and the TreeMap shows the combination of keywords; to better visualize the findings, we utilize the size of keywords to reflect the frequency of keyword occurrence (Figure 6C). Figure 6D’s tree map, on the other hand, depicts the link between the hierarchical order and the keywords discovered by the hierarchical clusters. The graphic depicts the connections between clusters and the items they include, revealing that each object consists of a succession of keywords associated with “electroactive materials” and “spinal cord damage.” Multiple correspondence analysis was utilized to develop the concept structure diagram (Figure 6E). We discovered that the keywords are divided into two major clusters, with the red cluster focusing on the treatment of spinal cord injury and the blue cluster focusing on the recovery of function after spinal cord injury, thereby providing researchers with a concise overview of research hotspots in this field. Figure 6F depicts the top 25 keywords in the keyword emergence. This finding indicates that the research hotspots from 2012 to 2014 were Schwann cells, but the present research hotspots are mostly focused on surgery, carbon nanotubes, exercise, the individual, and validity, offering researchers with further study paths.

### 3.7 Qualitative analyses

After applying the bibliometrix filter to papers with an average yearly citation of 10 or more, we retrieved 58 highly cited publications, comprising 27 reviews (46.55%) and 31 articles (53.0%). The qualitative analysis of highly cited literature is important for elucidating the research development in the topic and immediately grasping the research foundation. Based on particular study goals, we subdivided these 58 publications into three subcategories: genesis of neuronal function impairments, materials for neuroregenerative repairation, and relative mechanisms.

#### 3.7.1 Genesis of neuronal function impairments

**FIGURE 7:**
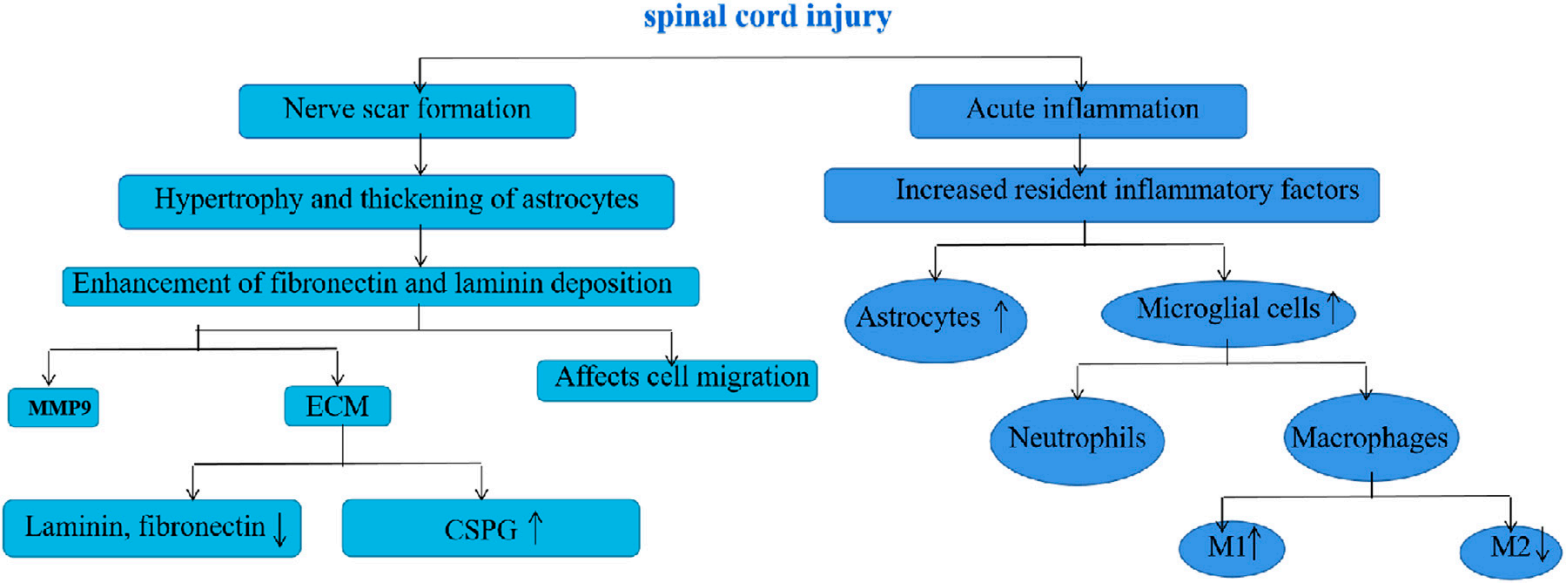
Specific mechanisms of spinal cord injury.

Utilizing biomaterials to treat spinal cord injuries needs a comprehensive understanding of the injury’s origin. Neuronal dysfunction is the major reason for reduced function in spinal cord injury. The primary causes of neuronal dysfunction include neural scar development, neuroinflammation in the injured spinal cord region, and axonal regeneration blockage resulting from apoptosis and necrosis (Liu X. et al., 2021; Kiyotake et al., 2022) (Figure 7). After nerve injury, astrocytes thicken, hypertrophy, and enhance the deposition of fibronectin and laminin, resulting in nerve scarring, according to a 2018 article with 210 citations; meanwhile, acute inflammation at the site of spinal cord injury increases astrocytes and microglia, recruiting neutrophils and macrophages, which affects the M1 and M2 polarization of myeloperoxidase and macrophages, and the different differentiation (Orr and Gensel, 2018). This is consistent with the findings of Brown, B.N. The involvement of macrophages in illness and post-injury tissue remodeling is dependent on their milieu, and M2-type macrophages can stimulate local tissue remodeling to some degree (Brown et al., 2012). In the early phases of injury, microglia safeguarded neural tissue viability by phagocytosing injured and degenerating tissue, while macrophages of peripheral origin were less phagocytic and their *in situ* mortality predisposed them to secondary injury in the central nervous system (Greenhalgh and David, 2014). In addition, the function of astrocytes following spinal cord damage is rather debatable. Several studies suggest that astrocytes may be able to heal early damage in the early stages of multiple sclerosis, whereas fibrillated extracellular matrix components, for instance, may facilitate axonal regeneration and recovery following central nervous system injury (Brosnan and Raine, 2013; Okada et al., 2018).

#### 3.7.2 Materials for neuroregenerative repairation

##### 3.7.2.1 Hydrogels

14 (24.14%) of the 58 highly referenced publications screened using bibliometrix involved the use of hydrogels for spinal cord injury healing. Since most spinal cord injuries do not affect the dura mater, implantation of a prepared scaffold, for instance, would cause subsequent damage to the patient. Injectable hydrogels may be loaded with cells and growth factors, and their mechanical characteristics can be altered (Aurand et al., 2012). In addition, injectable hydrogel-loaded medications are more easily able to traverse the blood-brain barrier, minimize cell aggregation, and create an adhesive matrix to enhance cell survival and integration (Macaya and Spector, 2012; Pakulska et al., 2012; Chedly et al., 2017). Accelerated conversion of neural stem cells (NSC) to neurons and suppression of astrocyte formation were achieved by utilizing a conductive, biocompatible hydrogel (CPH) constructed from tannic acid (TA), polypyrrole (PPy) chains (Zhou et al., 2018). In the same year, Rao et al. (2018) injected chitosan-loaded neurotrophin 3 (NT3) into the interspinal space of adult rhesus monkeys with a 1-cm resection, and motor axons in the corticospinal tract were able to traverse the lesioned area into the distal part of the spinal cord to recover motor and sensory functioning. However, hydrogel materials have limited precision and complexity in their geometry construction. Rapid gelation (within 20 s) and spontaneous covalent cross-linking for optimal formation of neural networks, promoting axonal regeneration and reducing glial scar deposition are made possible by 3D bioprinting technology using functional chitosan, hyaluronic acid derivatives, and matrix gels as bioinks. This opens up a new area of study for regenerative medicine (Liu N. K. et al., 2021).

##### 3.7.2.2 Self-assembled peptide scaffold

An article from 2012 with 387 citations describes the promising future of self-assembled peptide scaffolds in regenerative medicine (Matson and Stupp, 2012). As a type of biomaterial, self-assembled peptides are a collection of short peptides or peptide derivatives that exhibit enhanced biocompatibility, cellular signaling, and electrical signaling capabilities (Matson and Stupp, 2012). To further tailor the peptide backbone, these scaffolds can include cell-specific adhesion peptides, growth factors, and enzyme cleavable sequences. Extracellular matrix (ECM)-inspired three-dimensional (3D) structures have the potential to serve as cell culture models for studying the interplay between ECM cells and, ultimately, as templates for the regeneration of specific target tissues or organs (Ravichandran et al., 2014).

##### 3.7.2.3 Electrostatic spinning

To stimulate the differentiation of neural stem cells into motor neurons, Binan et al. (2014) created a disordered material composed of co-electrospun fibers of poly-L-lactic acid and gelatin with good degradation rate and mechanical characteristics, slow-release retinoic acid (RA), and genistein. Moreover, the electrostatic spinning-prepared material has piezoelectric capabilities, and the scaffold may suppress apoptosis, inflammation, and nerve scar formation, all of which promote neurogenesis, axonal development, and angiogenesis (Liu et al., 2012; Liu et al., 2019; Kiyotake et al., 2022).

#### 3.7.3 Restoration of nerve function after spinal cord injury using biomaterials

Repair of spinal cord nerve damage by biomaterials was the subject of seven papers (12.07%) with yearly citation rate of 10 or greater from 2012 to 2022. The expression of Caspase-3 and Bax was reduced, while the expression of Bal-2 was elevated, all of which contributed to polydation’s anti-oxidative stress effects and prevention of cell death (Apoptosis) (Lv et al., 2019). Decellularized spinal cord matrix hydrogels were shown to influence cell proliferation and migration by activating integrins Itg2, Itg9, and Itg1, and by boosting AKT/ERK phosphorylation, according to a study published by Xu et al. (2021). Both pro-inflammatory and restorative forms of differentiation are influenced by the direction in which macrophages are polarized in the surrounding microenvironment. It has been observed that 7–14 days after implantation, M1 to M2 cell populations may be converted by the extracellular matrix (ECM) scaffold material, and that the biomaterial’s surface topography can result in a considerably greater ratio of M2/M1 macrophages (Brown et al., 2012).

## 4 Discussion

Quantitative and qualitative analyses of 1,330 papers in the field of electroactive materials spinal cord injury in the WOS core database between 2012 and 2022 were performed in this study.

Quantitative study demonstrated an increasing pattern of both the total number of publications and the average yearly citation rate in the subject. The United States, China, and Canada account for the majority of authors, with the former playing a significant role in global collaboration. China’s publishing output has been on the rise since 2018, although the country’s citation count (4,556) remains lower than that of the United States’ (6,241). This points to China’s quick development in the field, but it also shows that the United States is the source of the best papers. Zhang Y has published the most papers (24) and has the greatest author impact score, both of which are indicative of highly-respected work in this area. The examination of search terms showed that the focus of medical research has shifted from Schwann cells, fractures, and urologic problems to carbon-based materials, functional recovery, and surgery. A lot of attention has been paid to studying the process of surgery and the subsequent functional recovery (Kunze and Bohn, 1978; Myers et al., 2019). In addition, lumbar and hip fractures are prone to spinal cord injuries, urinary system abnormalities, such as urine incontinence, and other frequent consequences (Ginsberg et al., 2012; Myers et al., 2019). Chwann cells (SCs), the myelinating glial cells of the peripheral nervous system, are neuroprotective, decrease cavitation degeneration following spinal cord nerve damage, and promote axon and myelin production (Cerqueira et al., 2018). However, the survival rate of SCs following transplantation into the location of spinal cord damage is exceedingly low, necessitating the identification of a suitable carrier to assure both cellular and drug action. Carbon-based materials with superior biocompatibility, electrical conductivity, and the capacity for regulated, gradual medication release have risen to the forefront of contemporary research (Kiyotake et al., 2022).

In the qualitative study, 58 articles with an annual citation rate >10 were examined. Based on the examination of citation frequency and publication time, we determined that the role and mechanism of injectable conductive hydrogels in spinal cord healing is an attractive area of study. Moreover, the most researched processes include oxidative stress (Nrf2) and apoptosis (Casepase 3). With further development of conducting biomaterials and study into the underlying processes, it will be feasible to perform a functional and full healing of bone marrow injuries.

Conductive biomaterials mainly include conductive polymers (CPs), piezoelectric materials, and carbon-based nanomaterials. Piezoelectric materials and carbon-based nanomaterials are characterized by ultra-thin film structures with large surface areas, high adhesion levels, and tunable mechanical properties, which can be used as electrical or nanocarriers for synaptic modulation, reduction of neuroinflammation, regulation of stem cell fate and repair of damaged neural cells/tissues (He et al., 2022); Nanowire-based degradable piezoelectric material driven by ultrasound (US) accelerates motor recovery, promotes neural stem cell differentiation and endogenous angiogenesis (Chen et al., 2022). However, a single thin film material cannot repair cystic cavities in the CNS caused by trauma or vascular injury, which prevent cell survival and axonal regeneration within the cavity (Hong et al., 2017). Hydrogel has excellent biocompatibility, injectability, etc., can fill the cystic cavity, guide the regeneration of axons and repair the neuroinflammatory microenvironment; using iron tetrasulfide, carboxymethyl chitosan and gold in a magnetic field to obtain anisotropy and through the release of hydrogen sulfide can induce longer axon formation a nd functional recovery of neural stem cells while inhibiting the expression of pro-inflammatory factors (Wang et al., 2023); In addition, injection of IKVAV-functionalized PA hydrogel at the injury site can promote axonal regeneration while promoting axonal regeneration, while the slow-release brain-derived neurotrophic factor (BDNF) of this hydrogel can reduce astrocyte proliferation after injury and exert axonal protection, thus promoting recovery after SCI (Hassannejad et al., 2019a; Hassannejad et al., 2019b). Therefore, the joint application of ultra-thin film structures with conductive polymers such as hydrogels to construct composites with better biocompatibility and mechanical properties may be one of the future research trends.

This research has some drawbacks. First, this study was confined to a quantitative and qualitative examination of articles in the WOS core database between 2012 and 2022, and the sample size of the data was a bit less. Second, qualitative analysis is more subjective than quantitative analysis, and various observation perspectives may lead to divergent findings. This work just reflects the current trends in the field of spinal cord damage with electroactive materials, provides a theoretical foundation, and suggests future research possibilities.

## 5 Conclusion

In recent years, carbon-based nanomaterials and functional recovery after spinal cord damage have been among the most popular study issues in this sector. Although many current studies using small animal SCI models for cell-based tissue engineering have yielded the expected results, to date, they have not been successfully put into clinical trials, and there is no completely curative cure available; recovery of the functional science associated with spinal cord injury is critical. This paper is an important resource for understanding and predicting the future of research on electroactive materials for spinal cord injury, as well as for guiding neural repair and regeneration following spinal cord injury.

## Data Availability

The original contributions presented in the study are included in the article/supplementary material, further inquiries can be directed to the corresponding authors.
